# Biochemical purification and characterization of a truncated acidic, thermostable chitinase from marine fungus for N-acetylglucosamine production

**DOI:** 10.3389/fbioe.2022.1013313

**Published:** 2022-10-04

**Authors:** Bin He, Liyan Yang, Dengfeng Yang, Minguo Jiang, Chengjin Ling, Hailan Chen, Feng Ji, Lixia Pan

**Affiliations:** ^1^ School of Animal Science and Technology, Guangxi University, Nanning, Guangxi, China; ^2^ State Key Laboratory of Non-Food Biomass and Enzyme Technology, Guangxi Key Laboratory of Marine Natural Products and Combinatorial Biosynthesis Chemistry, Guangxi Academy of Sciences, Nanning, Guangxi, China; ^3^ Guangxi Key Laboratory for Polysaccharide Materials and Modifications, School of Marine Sciences and Biotechnology, Guangxi University for Nationalities, Nanning, China; ^4^ Nanning Dabeinong Feed Technology Co., Ltd., Nanning, Guangxi, China; ^5^ Guangxi Huaren Medical Technolgoy Group, Nanning, Guangxi, China

**Keywords:** chitin, acidic chitinase, N-acetylglucosamine, AlphaFold2, N-terminal truncation, Aspergillus fumigatus, β-N-acetylglucosaminidase

## Abstract

N-acetylglucosamine (GlcNAc) is widely used in nutritional supplement and is generally produced from chitin using chitinases. While most GlcNAc is produced from colloidal chitin, it is essential that chitinases be acidic enzymes. Herein, we characterized an acidic, highly salinity tolerance and thermostable chitinase *Af*ChiJ, identified from the marine fungus *Aspergillus fumigatus* df673. Using AlphaFold2 structural prediction, a truncated Δ30*Af*ChiJ was heterologously expressed in *E. coli* and successfully purified. It was also found that it is active in colloidal chitin, with an optimal temperature of 45°C, an optimal pH of 4.0, and an optimal salt concentration of 3% NaCl. Below 45°C, it was sound over a wide pH range of 2.0–6.0 and maintained high activity (≥97.96%) in 1–7% NaCl. A notable increase in chitinase activity was observed of Δ30*Af*ChiJ by the addition of Mg^2+^, Ba^2+^, urea, and chloroform. *Af*ChiJ first decomposed colloidal chitin to generate mainly N-acetyl chitobioase, which was successively converted to its monomer GlcNAc. This indicated that *Af*ChiJ is a bifunctional enzyme, composed of chitobiosidase and β-N-acetylglucosaminidase. Our result suggested that *Af*ChiJ likely has the potential to convert chitin-containing biomass into high-value added GlcNAc.

## Introduction

Chitin is homopolymer consisting of β-1, 4-N-acetyl-D-glucosamine (GlcNAc) units in a linear form (Chen et al., 2021). It exists in the skeletal organic matrices of coraline algae (Rahman and Halfar, 2014) and in the extracellular matrix of various invertebrates, including sponges, molluscs, nematodes, arthropods, and fungi (Lambris, 2021). Chitin is also a white, hard, inelastic polysaccharide and is a substantial contributor to pollution in coastal zones (Kumar, 2000). It is commonly thought that chitin is the second most plentiful and renewable polysaccharide on earth, after cellulose (Duo-Chuan, 2006; Yang et al., 2009). At least 10 GT chitin is synthesized and decay each year in the ecosystem (Jolles et al.). Approximately 10^10^–10^12^ kg chitin are yielded yearly by living organisms in seas and oceans (Dhillon et al., 2013), but decomposing chitin under natural environmental conditions is extremely tricky. However, this massive quantity of chitin would supply gigantic amounts of raw material. However, the currently huge amounts of chitin-containing waste produced by seafood processing industries is burned, landfilled, discarded at sea, or left to decay (Xu et al., 2013). This may cause environmental stress and is a waste of raw chitinous materials.

The conversion of chitin into derivatives, i.e., chito-oligosaccharides (COS), N-acetylglucosamine, and chitosan, exhibits a tremendously wide range of biological properties and outstanding potential applications, such as in the medicine and cosmetics (Jimtaisong and Saewan, 2014; Kumar et al., 2015), agriculture and aquaculture (Razdan and Pettersson, 1994; Wang and Chen, 2005), and materials science and membranes (Wang et al., 2016; Chen et al., 2018). GlcNAc, the terminal hydrolysis of chitin, is a crucial functional amino sugar complex that could serve as healthcare, medicine, and skin care products with a large requirement. Consequently, it may be possible to convert inexpensive chitin into high-value GlcNAc.

The enzymatic hydrolyzation of chitin-containing debris has been suggested as a cost-effective and sustainable way of producing GlcNAc (Liu et al., 2020). Chitinase (EC 3.2.1.14) is a type of glycosyl hydrolase enzyme that cleaves the bond between the C1 or C4 of two successive N-acetyl-β-D-glucosamine molecules in chitin (Xia et al., 2001; Hou et al., 2020). Chitinases are classified into two major groups, namely, endochitinase (EC 3.2.1.14) and exochitinase (EC 3.2.1.29). Endochitinases randomly hydrolyze chitin at internal sites to generate multimers of GlcNAc (Saima et al., 2013). Exochitinases contain two subcategories, called chitobiosidases (EC 3.2.1.29) and β-N-acetylglucosaminidases (EC 3.2.1.52). Chitobiosidases can progressively liberate (GlcNAc)_2_, beginning at the nonreducing end of the chitin. β-N-acetylglucosaminidases can crack the oligomeric products of endochitinases and chitobiosidases, further converting them to monomer GlcNAc (Wang and Wu, 2020). The thorough hydrolyzation of chitin for GlcNAc production requires the combining action of endochitinase and β-N-acetylglucosaminidases or chitobiosidases and β-N-acetylglucosaminidases (Du et al., 2019). The assembly of different chitinases of *Sm*ChiA, *Sm*ChiB, and *Sm*ChiC from the microorganism *Serratia marcescens* and a β-N-acetylglucosaminidases of *Of*Hex1 from the insect *Ostrinia furnacalis* have been used to yield GlcNAc from the mycelial product in the processing waste of the fermentation industry (Zhu et al., 2016). However, the isolation of various chitinases from the cytoplasm of *E. coli* for industrial application is costly and difficult. For this reason, an enzyme that combines the activity of endochitinase and NAGase or chitobiosidases and NAGase would be much more suitable for industrial application.

Chitin has a highly organized crystalline structure, consisting of powerful intermolecular hydrogen bonding and intramolecular hydrogen bonding networks, such that this hydrogen bonding contributes to the strength and rigidity of the chitin chain (Cohen, 2001; Khan et al., 2017). Chitin has three polymorphic isomers viz. α-chitin, β-chitin, and γ-chitin. Among these, α-chitin is the most common form in nature, and α-chitin decays more slowly than the other two kinds, as it has higher recalcitrance, which decreases the accessibility of the chitin chains. Chitin is insoluble in water and most inorganic and organic solvents, except for concentrated acid (Hou et al., 2019). Colloidal chitin is usually produced by processing chitin with concentrated acids to destroy the crystalline structure and enhance the approach of chitin to chitinase and lytic polysaccharide monooxygenases. Therefore, colloidal chitin is commonly applied as the substrate for chitinase to prepare COSs and GlcNAc (Ren et al., 2022). Meanwhile, resources of ocean-derived chitin contain about 3% NaCl. Thus, the stability of chitinases at certain concentrations of salt, high temperature, and low pH can magnify their commercial utilization relative to their salt-free environment and their mesophilic and neutrally stable counterparts using the raw materials of colloidal chitin collected from ocean debris.

Marine chitinases possess higher pH and salinity tolerance than chitinases from terrestrial organisms (Han et al., 2009). These chitinases exist in the marine bacteria *Pseudoalteromonas flavipulchra* DSM 14401 (Ren et al., 2022) and the *Paenibacillus* strain UMBR 0002 (Liu et al., 2020). Researchers have identified chitinases produced by more than 30 marine bacteria (Xinyue et al., 2021), but few studies have investigated chitinase in marine fungus. Further, many thermostable chitinases have been discovered from fungi (Krolicka et al., 2018). The genomes of the fungal *Aspergillus fumigatus* are well known to be rich in chitinase genes. This species contains 17 chitinases at least, including 5 class III chitinases. Class III chitinases are normally acidic and may play a nutritional role during autolysis (Alcazar-Fuoli et al., 2011). Nevertheless, the majority of previous reports on *A. fumigatus* have focused on terrestrial organisms (Xia et al., 2009; Wang et al., 2016). Herein, we adopted *A. fumigatus* df673 as a research strain, stored in our lab, previously obtained from the tidal flat sediments of mangrove in Beibu Gulf, Guangxi Province, China. To date, little research has reported on the chitinases of oceanic *A. fumigatus*. The analysis of the marine *A. fumigatus* genome is hoped to extract thermostable chitinases with acid-resistance, highly salinity tolerance, and high enzyme activity adapted to industrial applications. Herein, we successfully cloned and expressed the chitinases *Af*ChiJ, Δ19*Af*ChiJ, and Δ30*Af*ChiJ with an *E. coli* BL21 (DE3) expression system. The N-truncated chitinase of Δ30*Af*ChiJ was successfully purified to homogeneity and biochemically characterized. *Af*ChiJ is acid-resistant, has a high salinity tolerance, and is a thermostable exochitinase, a GH18 chitinase belong to the fungal/plant class chitinase (class III) family of filamentous fungi (Alcazar-Fuoli et al., 2011). *Af*ChiJ degrades colloidal chitin to generate mainly N-acetyl chitobioase at first, which is further hydrolyzed to GlcNAc. The results suggest that the marine chitinase *Af*ChiJ is an exochitinase that contains the properties of both chitobiosidases and β-N-acetylglucosaminidase. It is concluded that *Af*ChiJ is a promising and sustainable option for GlcNAc preparation using chitin-containing biomass resources.

## Materials and methods

### Materials

We obtained QuickCut *Nco*I, QuickCut *Nde*I, and QuickCut *Xho*I restriction endonucleases and a recombinant DNase I (RNase-free) and PrimeScript II 1st Strand cDNA Synthesis Kit from TaKaRa (Dalian, China). The FastPure Gel DNA Extraction Mini Kit, DNA polymerases and 2 × ClonExpress Mix were all purchased from Vazyme Biotech CO., Ltd. (Nanjing, China). StarPrep Plasmid Miniprep Kit, Direct-load Color Prestained Protein Marker (15–150 kDa), Direct-load Star Marker D2000, and Star Marker D5000 were purchased from GenStar (Beijing, China). Host cell strains, *E. coli* DH 5α, *E. coli* BL21 (DE3), and the pET-22b (+) were stored the lab in Guangxi Academy of Sciences. Chitin, chitosan, cellulose microcrystalline, sodium carboxymethylcellulose (CMC-Na), and N-acetyl-D-glucosamine (GlcNAc) were purchased from BBI Co. Ltd. (Shanghai, China). Chitin oligosaccharides with degrees of polymerization (DP) between 1 and 6 were purchased from Tokyo Chemical Industry Co. Ltd. (Tokyo, Japan). Ni NTA Beads 6FF was purchased from Smart-Lifesciences Biotech (Changzhou, China). Redprussiate, isopropyl-beta-D-thiogalactoside (IPTG), aniline, and diphenylamine were purchased from 9dingchem (Shanghai, China). All other reagents were of analytical grade or the guaranteed grade available unless otherwise mentioned.

### Strains and culture conditions

*Aspergillus fumigatus* df673 had previously been isolated by our lab from tidal flat sediments of mangrove in Beibu Gulf, Guangxi Province, China. It was stored in the Guangxi Beibu Gulf Marine Research Center and was cultured with potato dextrose broth medium at 28°C. *E*. *coli* strains were grown in LB culture medium (10 g tryptone, 5 g yeast extract, and 10 g sodium chloride per liter) at 37°C (1.5% agar powder should also be added to the solid medium).

### Chitinous substrates

α-Chitin was obtained from crab shells (BBI; Shanghai, China) and then ball-milled by the inverter planetary ball mill XQM-2L (Kexi, Nanjing, China). The chitin powder was ground at 300 rpm for total of 9 h with 3 h grinding cycles interrupted 1 h pauses, producing an ultimate average particle size of 0.15 mm (Mekasha et al., 2020).

β-Chitin was acquired from a squid pen with an average particle size of 0.3 mm. The squid pen was collected from Nanning seafood wholesale trading market (Lecun Road 4, Nanning, Guangxi Province) and stored at −20°C until being handled in the lab. β-Chitin was prepared according to (Lavall et al., 2007): the squid pens were repeatedly washed with water, dried at 50°C, and then ground to particulates of less than 0.3 mm by the inverter planetary ball mill XQM-2L. In all, 1 g powder was dissolved in 10 ml hydrochloric acid (0.55 M) at 25°C for 2 h, and the treatments were repeated. Then, 1 g product was dissolved in 20 ml sodium hydroxide (0.3 M) at 80°C for 1 h, in two sequential treatments (Tolaimate et al., 2000). Finally, the product was washed to neutral with double distilled water, dried at 80°C, and stored at −40°C for following use.

Colloidal chitin was prepared as previously described by Wu H et al. (2022). Briefly, 20 g chitin (Aladdin, Shanghai, China) was stirred in 200 ml concentrated phosphoric acid (4 M) at room temperature for 2 h and then placed at 4°C for 24 h. Subsequently, 2 L cooled double distilled water and gelatinous chitin were mixed and placed for 24 h at 4°C. The colloidal chitin was separated by centrifugation at 6,000 rpm for 15 min and washed to neutral with double distilled water. Finally, the colloidal chitin was dissolved in 1 L phosphate-citrate buffer (pH = 7) to an ultimate concentration of 2%.

### Enzyme assay

The activities of the purified recombinant Δ30*Af*ChiJ and crude proteins toward the substrate were assayed according to the Schales method with some subtle modifications (Imoto and Yagishita, 1971). To detect the chitinase activity, the reaction containing 100 µL enzyme solution, 400 µL substrate (at a ultimate concentration of 2%) dissolved in phosphate buffer was incubated for 30 min at 45°C. The reaction was stopped by the addition of 1,000 µL Schales buffer and heating for 5 min in boiling water. The optical density (OD) of the supernatant was assayed at 420 nm using the Infinite M200 pro microplate reader (TECAN, Switzerland). The chitinase activity was determined by colorimetry and defined as the amount of enzyme that produces 1 µmol GlcNAc from the substrate per min.

### Isolation of total RNA from *A. fumigatus* df673 and reverse transcribed to cDNA

*A. fumigatus* df673 was cultured in conical flask with potato dextrose broth (PDB) medium in a constant temperature incubator of 200 rpm for 3 days at 28°C. Total RNA was isolated from *A. fumigatus* df673 with Trizol solution (Invitrogen). The degradation of DNA was performed in the isolated total RNA samples (20 µg) using Recombinant DNase I (RNase-free) (Takara, Japan). The total RNA was reverse transcribed to cDNA using a PrimeScript II 1st Strand cDNA Synthesis Kit (Takara, Japan). The kit instructions were followed for all steps. cDNA was stored at −20°C (Reddy and Sarma, 1995).

### Bioinformatic analyses

The analysis and searches of sequenced data were carried out by NCBI. Signal peptides of the chitinases were predicted by SignalP 5.0 (https://services.healthtech.dtu.dk/service.php?SignalP-5.0) (Almagro Armenteros et al., 2019). The domain of the chitinases were predicted on the PROSITE website (https://prosite.expasy.org) (Hulo et al., 2006). The generated sequences from the current study and reference sequences from Cazy (http://www.cazy.org) were used to create a phylogenetic tree using the neighbor-joining method (NJM) with 2000 replications for each bootstrap value, using the MEGA 11 software version (Tamura et al., 2021). Multi-sequence alignment results were visualized using the ENDscript 2.0 (https://endscript.ibcp.fr/ESPript/ENDscript/). The three-dimensional structure of *Af*ChiJ was predicted by AlphaFold2 (Cramer, 2021). Ligand molecule (GlcNAc)_4_ was drawn using ChemDraw software. Molecular docking was performed by AutoDock 4.0 (Morris et al., 2009) and flexible docking methods. PyMOL (https://pymol.org/) (Rigsby and Parker, 2016) was carried out visualization of the modeled structure and the analysis of detailed interactions.

### Cloning of the chitinase genes and design of the recombinant plasmid

The chitinase (*Af*ChiJ, Δ19*Af*ChiJ, Δ30*Af*ChiJ) genes were amplified using the primer sets listed in Table 1. The nucleotide sequence of *Af*ChiJ gene was uploaded in GenBank with the accession number: ON932820. cDNA of *A. fumigatus* df673 was used as the PCR template. PCR products were cleaned with PCR clean-up kit according to manufacturer’s description. The purified *Af*ChiJ fragment was ligated into pET-22b (+) that was digested with *Nde* I and *Xho* I by ClonExpress II One Step Cloning Kit (Vazyme, China), generating the recombinant plasmid named pET-22b (+)-*Af*ChiJ. Other expression plasmids pET-22b (+)-Δ19*Af*ChiJ and pET-22b (+)-Δ30*Af*ChiJ were constructed by the same strategy. The plasmids pET-22b (+)-*Af*ChiJ, pET-22b (+)-Δ19*Af*ChiJ, and pET-22b (+)-Δ30*Af*ChiJ were respectively transformed into expression host BL21 (DE3).

**TABLE 1:** Primer sequences used to amplify the chitinase gene.

Infusion cloning primers	Sequence 5′ to 3′
**Δ30*Af*ChiJ** forward primer	CCC​AGC​CGG​CGA​TGGCCA​TGGATG​CCC​AGA​ACG​TTG​TGT​ACT​G
**Δ30*Af*ChiJ** reverse primer	GTG​GTG​GTG​GTG​GTGCTC​GAGACA​AGG​AGA​CCC​GGT​AGT​CAG​G
**Δ19*Af*ChiJ** forward primer	CCC​AGC​CGG​CGA​TGGCCA​TGGATC​TGC​CCC​ATC​AGC​TAT​CTT​C
**Δ19*Af*ChiJ** reverse primer	GTG​GTG​GTG​GTG​GTGCTC​GAGACA​AGG​AGA​CCC​GGT​AGT​CAG​G
***Af*ChiJ** forward primer	TAA​GAA​GGA​GAT​ATACAT​ATGTAT​TTT​ACC​ACA​TTG​CTC​AGT​GCC
***Af*ChiJ** reverse primer	GTG​GTG​GTG​GTG​GTGCTC​GAGACA​AGG​AGA​CCC​GGT​AGT​CAG​G

Underlining indicates the added *Nco*I & *Xho*I, *Nco*I & *Xho*I, and *Nde*I & *Xho*I sites.

### Expression and purification of chitinase

The recombinant plasmids pET-22b (+)-*Af*ChiJ, pET-22b (+)-Δ19*Af*ChiJ, and pET-22b (+)-Δ30*Af*ChiJ were respectively transformed into the expression host BL21 (DE3) to express chitinase. Cells were grown at 37°C to OD_600_ about 0.6–1.0, then induced by adding IPTG to ultimate concentration of 0.1 mM, and the cells were cultured for 8 h at 25°C. The cell pellet was separated by centrifugation (Avanti J-26S XP, Beckman Coulter, United States) at 7,000 rpm for 10 min and resuspended in NaH_2_PO_4_-NaCl buffer (50 mM NaH_2_PO_4_, 300 mM NaCl, pH 8.0), then lysed on ice using an ultrasonic cell disrupter (Sonics VCX-750, SONICS, United States), and the cell fragments was precipitated by centrifugation at 4°C for 30 min (12,000 rpm). The supernatant was loaded onto a column containing Ni-NTA Beads 6FF (Smart-Lifesciences, Changzhou, China) for His-tag affinity purification. The column was washed with wash buffer (50 mM NaH_2_PO_4_, 300 mM NaCl, 25 mM imidazole, pH 8.0) to remove contaminating proteins. Chitinase was eluted with elution buffer (50 mM NaH_2_PO_4_, 300 mM NaCl, 250 mM imidazole, pH 8.0). The protein that was finally obtained was detected using 12% sodium dodecyl sulfate polyacrylamide gel electrophoresis (SDS-PAGE). The elution buffer was concentrated and desalted with a 10 kDa centrifuge ultrafiltration tube (Millipore, Massachusetts, United States) with 100 mM phosphate-citrate buffer (pH 7.0). The protein concentration of Δ30*Af*ChiJ was measured using the NanoDrop 2000 (Thermo Fisher Scientific, United States).

### Biochemical characterization of Δ30*Af*ChiJ

The optimal temperature for Δ30*Af*ChiJ activity was measured between 25–80°C in 100 mM phosphate-citrate buffer at pH 7.0 for 30 min. The thermal stability of Δ30*Af*ChiJ was studied at different temperatures from 25°C to 70°C substrate-free for 60 or 90 min, then the residual enzyme activity was detected according to the standard assay condition. The optimum pH of the Δ30*Af*ChiJ activity was determined using phosphate-citrate buffer (pH 2–8) and glycine-NaOH buffer (pH 8–10). Chitinase was incubated in above buffer at pH 1.0–11.0 at 30°C for 3 h to determine the remaining activity.

The influences of metal ions and chemical reagents (Ca^2+^, Co^2+^, Al^3+^, NH_4_^+^, Zn^2+^, Cu^2+^, K^+^, Ni^2+^, Na^+^, Mn^2+^, Li^+^, Mg^2+^, Ba^2+^, Tris, ethylenediaminetetraacetic acid (EDTA), sodium dodecyl sulfate (SDS), and urea) on Δ30*Af*ChiJ activities were measured in the presence of 10 and 50 mM; at the same time, the effects of organic solvents (methanol, ethanol, acetonitrile, ethyl acetate, chloroform, dimethylsulfoxide (DMSO), tween-20, tween-80, glycerin, and isopropyl alcohol) were measured at ultimate concentrations of 10 and 20% (v/v). Each compound was added to the standard enzyme reaction system and assayed under optimal conditions.

For the halotolerance assay, the purified chitinase of Δ30*Af*ChiJ was incubated with different ultimate concentrations of NaCl (1, 2, 3, 4, 5, 7, 9, or 11%) at 4°C for 3 h, and the residual Δ30*Af*ChiJ activity toward colloidal chitin was detected under optimum temperature and pH.

### Substrate specificity

The zymolyte specificity assay of the purified Δ30*Af*ChiJ was studied at 45°C in 100 mM phosphate-citrate buffer (pH 4) with zymolyte including α-chitin powder, β-chitin powder, colloidal chitin, cellulose microcrystalline, sodium carboxymethylcellulose (CMC-Na), and chitosan. Each compound was appended to the standard enzyme reaction system at an ultimate concentration of 2% (w/v) and assayed under optimal conditions with the highest activity, defined as 100%.

### Product assay

Reaction products from colloidal chitin hydrolyzed by Δ30*Af*ChiJ were inspected by silica gel thin-layer chromatography (GF254, Qingdao, China). The reaction mixture was put into effect with the basis of the way (Morimoto et al., 2001) with some subtle changes. The reaction containing 400 µL colloidal chitin (2%) and 100 µL Δ30*Af*ChiJ dissolved 100 mM phosphate-citrate buffer (pH 4) were incubated at 45°C for 1 h, 2 h, 3 h, 4 h, 6 h, and 8 h. The reaction mixturesysis was heated for 5 min in boiling water and centrifuged at 12,000 rpm for 15 min at 4°C. The supernatant was detected by TLC, which was spread out with a solvent of n-butanol, acetic acid, and aqueous (2:1:1). The products could be visualized with the method of spraying a diphenylamine-aniline-phosphate reagent (a blend of 1 ml aniline, 1 g diphenylamine, 5 ml concentrated phosphoric acid, and 50 ml acetone) evenly on the plate and heating the plate at 80°C for 20 min.

The main products of colloidal chitin degraded by Δ30*Af*ChiJ were determined by high performance liquid chromatography (HPLC, Thermo Fisher Scientific, United States). The reaction mixturesysis, including 400 µL colloidal chitin (2%) and 100 µL Δ30*Af*ChiJ dissolved in 100 mM phosphate-citrate buffer (pH 4) was incubated at 45°C for 1 h, 3 h, and 6 h. The reaction was stopped by adding 500 µL acetonitrile (75%) and then centrifuged at 12,000 rpm for 10 min at 4°C. After centrifugation, 10 µL supernatant was pushed into the HPLC system with a differential refractive index detector and a Luna NH_2_ column (250 mm × 4.6 mm, Phenomenex), at the temperature of 45°C. The mobile phase was acetonitrile: water (75:25, v/v), and its flow rate was 0.6 ml/min.

### Detect the activity of β-N-acetylglucosaminidases

β-N-acetylglucosaminidase activity of Δ30*Af*ChiJ was measured by breaking 4-methylumbelliferyl-GlcNAc (4MU-GlcNAc) zymolyte. The activity was calculated with a fluorometric assay based on detecting of chromophores of the released 4MU from chitinase-specific zymolyte (Guthrie et al., 2005). To determine the β-N-acetylglucosaminidases activity, a reaction mixture containing 5 µL enzyme solution and 95 µL 5 mM 4MU-GlcNAc dissolved in Mcllvaine buffer (pH 7.0) was incubated for 10 min at 45°C. The reaction mixture was stopped by the adding of 100 µL 1M glycine/NaOH buffer of pH 10.6 for readings and were taken immediately by use of a Fluorescence Spectrophotometer (HITACHI F-7000, Japan), the fluorescence was monitored (emission 460 nm, excitation 360 nm). β-N-acetylglucosaminidases activity was measured by 4MU released from 4MU-GlcNAc. The activity was counted by a standard curve of fluorescence units and 4MU (from 1 to 14 µM). One unit of β-N-acetylglucosaminidases activity was specified as the amount of enzyme which produces 1 µmol of 4MU from 4MU-GlcNAc per min (Mccret and Gooday, 1992).

## Results and discussion

### Bioinformatic analyses and construction of three constructs

**FIGURE 1:**
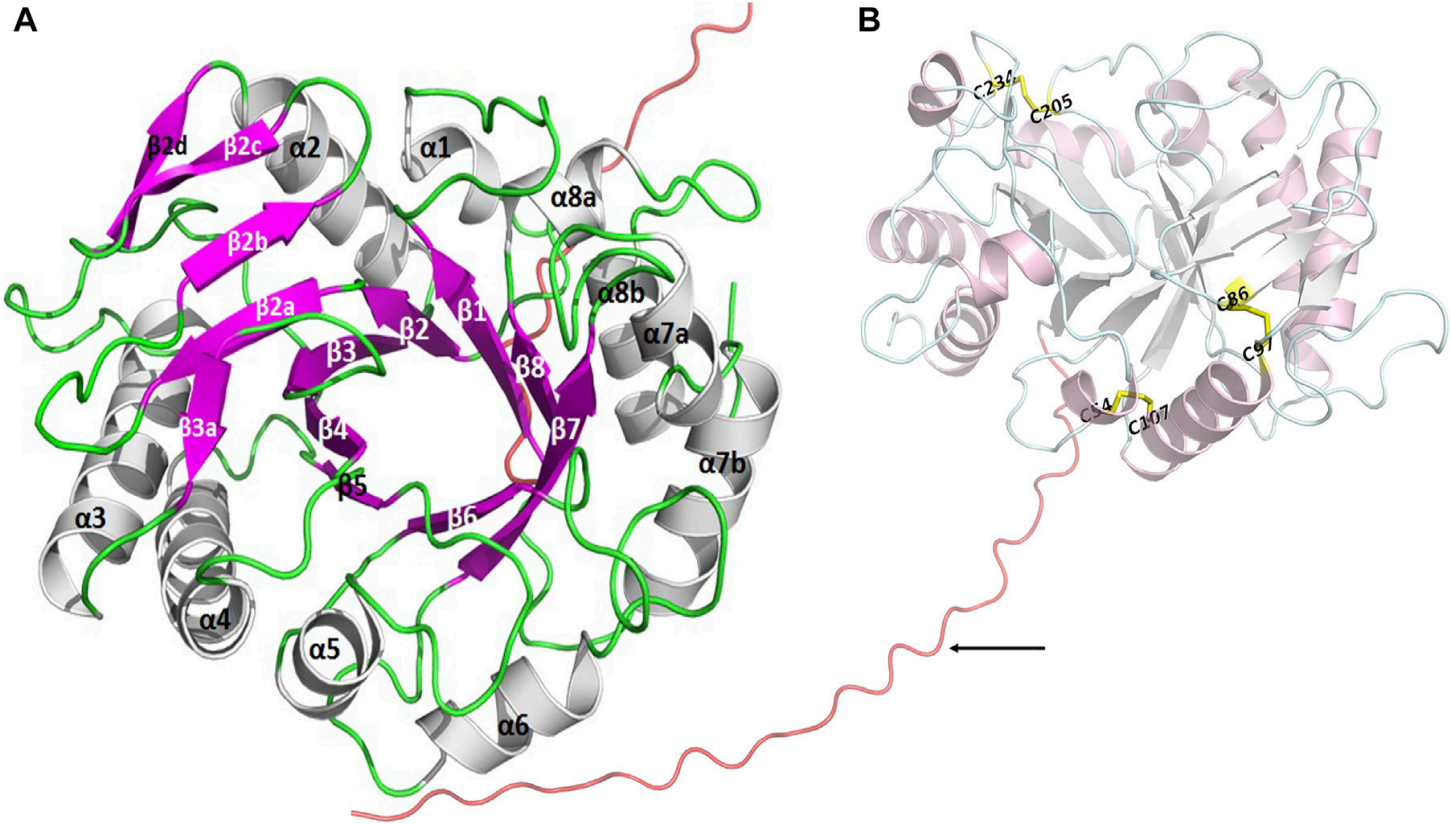
The tertiary structure of *Af*ChiJ was predicted by AlphaFold2. **(A)** *Af*ChiJ consisted of a (β/α)_8_ TIM barrel fold with an additional α/β structural domain, the additional α/β structural is composed of four antiparallel β-strands, inserted in the loop connecting helix α2 and barrel chain β2. **(B)** The N-terminal of *Af*ChiJ contained a flexible region of 30 amino acids in tv-red color, with the 30 amino acids being identified by black arrows. The three disulfide bridges C54–C107, C86–C97, and C205–C234 are shown in yellow.

**FIGURE 2:**
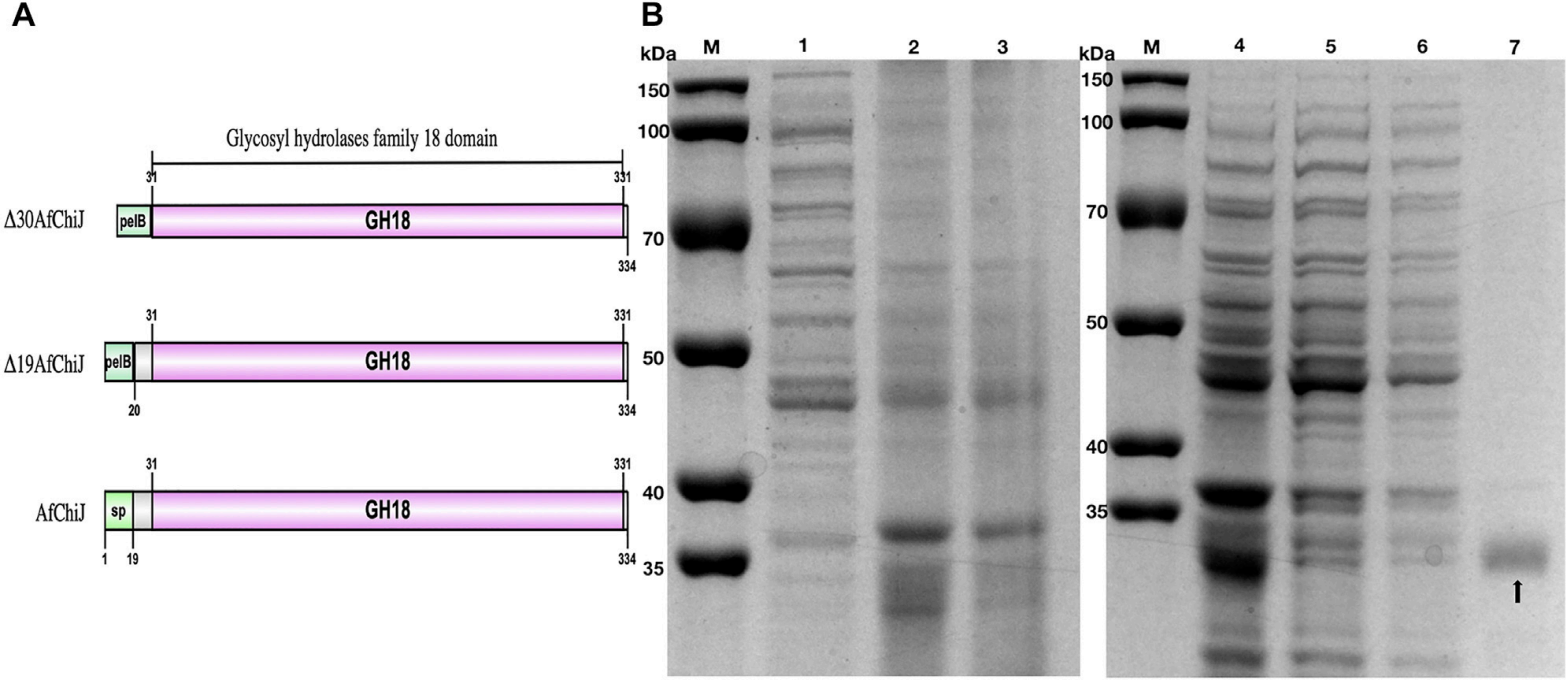
Schematic structure domains and SDS-PAGE analysis of chitinase Δ30*Af*ChiJ. **(A)** Schematic structure domains of *Af*ChiJ and its N-terminal truncation variants, Δ19*Af*ChiJ and Δ30*Af*ChiJ use signal peptide of pelB from pET-22b (+). **(B)** SDS-PAGE analysis of the purified chitinase Δ30*Af*ChiJ. M, protein molecular weight markers; 1, crude extract of *E. coli* BL21 (DE_3_)/pET-22b; 2, crude extract of *E. coli* BL21 (DE_3_)/pET-22b-Δ30*Af*ChiJ; 3, supernatant of *E. coli* BL21 (DE_3_)/pET-22b-Δ30*Af*ChiJ; 4, supernatant of *E. coli* BL21 (DE_3_)/pET-22b-Δ30*Af*ChiJ; 5, unbound protein through Ni-NTA Beads 6FF column; 6, protein eluted with buffer containing 25 mM imidazole; 7, protein eluted with buffer containing 250 mM imidazole. The gel was stained with Coomassie Brilliant Blue R-250.

A target gene band was produced by PCR from marine *A. fumigatus* df673, designated as *Af*ChiJ. The size of the PCR production was 1,002 bp (No. ON932820), which encoded 334 amino acids containing GH18 domains. The first 19 amino acids of *Af*ChiJ were determined as the signal peptide using the SignalP 5.0 analysis. The pH of *Af*ChiJ was 4.6, and the theoretical molecular mass was 35.4 kDa, calculated using the ExPASy Tool. The tertiary structure of *Af*ChiJ was predicted using AlphaFold2. The simulated structure indicated that *Af*ChiJ consisted of a (β/α)_8_ TIM barrel fold, accompanied by an additional α/β structural domain, formed of four antiparallel β-strands, inset in the loop connecting helix α2 and barrel chain β2 (Figure 1A). The 3 disulfide bridges in *Af*ChiJ were annotated in yellow, and the three-dimensional structure was stabilized by three disulfide bridges: C54–C107, C86–C97, and C205–C234 (Figure 1B). The homologue proteins previously reported were mostly expressed in *Pichia pastoris* (Landim et al., 2017). In this paper, we first try to use *E. coli* expression system with the high expression level of the target gene and short culture cycle to express *Af*ChiJ. Meanwhile, we choose a plasmid of pET-22b (+) with a signal peptide of pelB to express this enzyme because signal peptides can bring proteins into the periplasmic space of *E. coli*, which is conducive to the correct formation of protein disulfide bonds and improves the stability of protein. At the same time, the N-terminal of *Af*ChiJ contains a flexible region of 30 amino acids, as shown in tv-red color (Figure 1B). When *E. coli* is used to produce a eukaryotic protein, it is desirable to design the different constructs to produce as large an amount of the soluble and active protein as possible. With the aim of generating *Af*ChiJ constructs with increased stability and expression, we selected the N-terminal flexible region and signal peptide in *Af*ChiJ to test the influence of deleting them on protein expression and purification. Herein, we constructed three constructs in the schematic structure domains of chitinase (Figure 2A): a full length *Af*ChiJ with native signal peptide (1-334 AAs), N-terminal truncation 19 amino acids of Δ19*Af*ChiJ (20-334 AAs), and N-terminal truncation 30 amino acids of Δ30*Af*ChiJ (31-334 AAs). Δ19*Af*ChiJ and Δ30*Af*ChiJ used the signal peptide of pelB.

### Expression and purification of chitinases

Because the recombinant *Af*ChiJ mainly formed an insoluble protein, we calculated that the yield (total activity %) rate of *Af*ChiJ was only 8.51% after passing through the Ni-NTA affinity chromatography column. Nevertheless, a large amount of protein was precipitated when Δ19*Af*ChiJ was purified by passing through the Ni-NTA affinity chromatography column in 4°C. It was declared that Δ19*Af*ChiJ was extremely unstable in the process of purification. Finally, we failed to obtain purified Δ19*Af*ChiJ.

The yield rate of Δ30*Af*ChiJ reached 60.71%, and the purification was 69.98-flod (Table 2). Thus, we purified a large amount of Δ30*Af*ChiJ recombinant protein and proceeded to the next step of the research (Figure 2B). The 30 aa at the N-terminal of the chitinase of *Af*ChiJ neither formed a secondary structure nor a tertiary one, based on the AlphaFold2 structure prediction (Figure 1B). In this study, the N-terminal truncation of Δ30*Af*ChiJ can be successfully obtained from large amounts of solubly expressed protein. In a similar case, Fuciños et al. (2011) removed 16 or 26 aa from the N-terminal of esterase, which contributed to slight influences for substrate specificity. The results showed that the N-terminal fragment is pivotal for preserving the thermophilic property and stability, as well as the activity of the enzyme. Meanwhile, the ChiW protein was successfully overexpressed in *E. coli* by the method of N-terminally truncated (Itoh et al., 2014).

**TABLE 2:** Chitinase of Δ30*Af*ChiJ purification.

Purification step	Volume (ml)	Protein concentration (mg/ml)	Enzyme activity (mU/ml)	Specific activity (mU/mg)	Total activity (mU)	Purification (flod)	Yield % (total activity)
Crude enzyme solution	15	50	63.03	1.26	945.45	1	100
Ni-NTA affinity chromatography	7	0.93	82	88.17	574	69.98	60.71

### Effect of temperature and pH on Δ30*Af*ChiJ activity and stability

**FIGURE 3:**
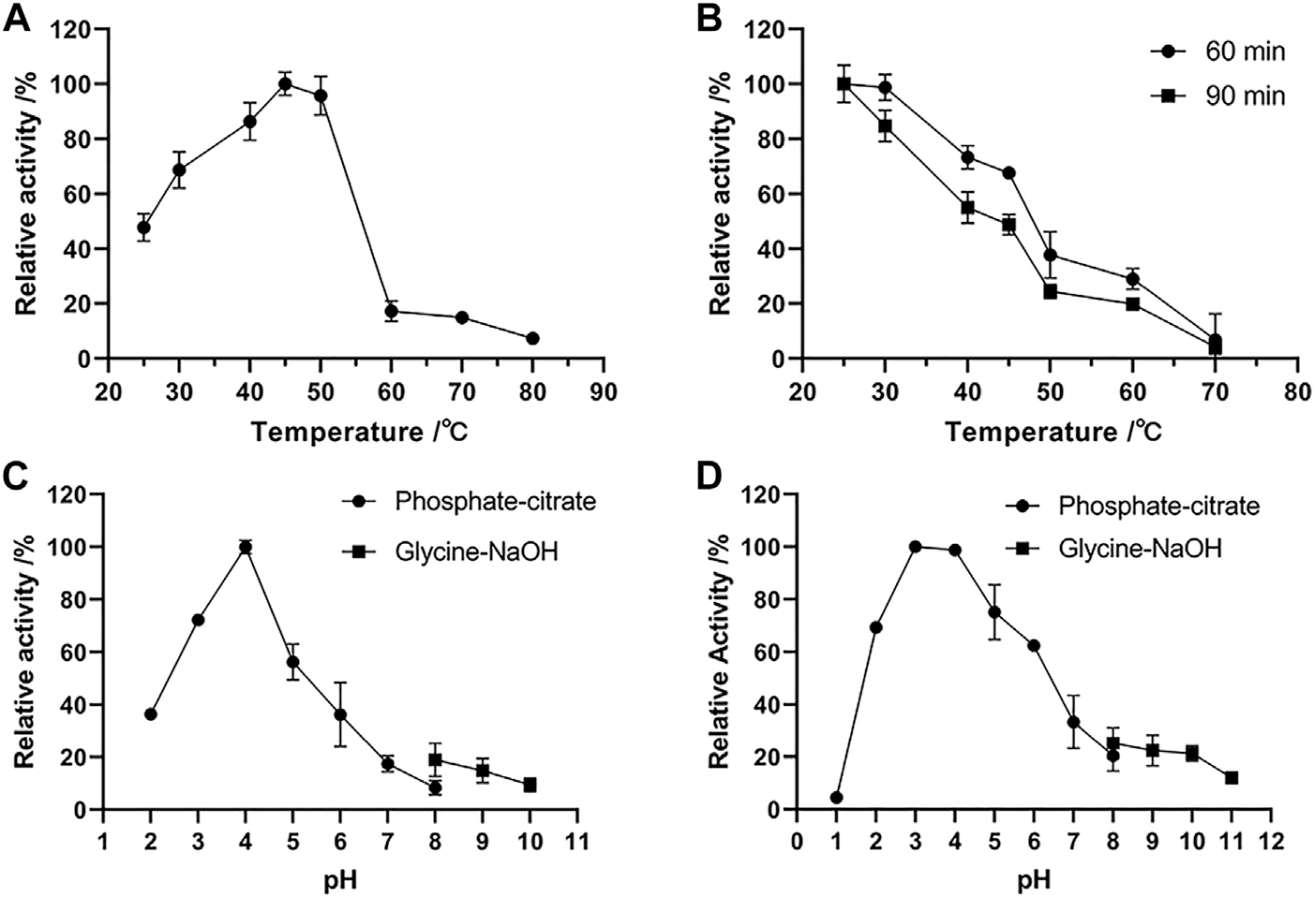
Effects of temperature and pH on the activity and stability of chitinase Δ30*Af*ChiJ. **(A)** The optimum temperature of Δ30*Af*ChiJ. The activity of Δ30*Af*ChiJ was measured at pH 7.0 with colloidal chitin as the substrate. The highest activity of Δ30*Af*ChiJ at 45°C was defined as 100%. **(B)** Thermostability of Δ30*Af*ChiJ. The thermostability was measured at pH 4.0. The highest enzyme activity after preincubation was defined as 100%. **(C)** The optimum pH of Δ30*Af*ChiJ. The activity of Δ30*Af*ChiJ was measured at 45°C with colloidal chitin as the substrate. The activity of Δ30*Af*ChiJ at pH 4.0 was defined as 100%. **(D)** pH stability of Δ30*Af*ChiJ. Stability of Δ30*Af*ChiJ after incubation at different pH at 30°C for 3 h without substrates. The activity of the enzyme after incubation at pH 3.0 was defined as 100%. Each data point represents the mean of three independent experiments, standard errors are shown (mean ± SD).

The optimum temperature of Δ30*Af*ChiJ activity was detected to be approximately 45°C by colloidal chitin as a zymolyte (Figure 3A). Δ30*Af*ChiJ remains much more than 68% of the Δ30*Af*ChiJ activity between 30 and 50°C. The activity of Δ30*Af*ChiJ decreased markedly above 50°C. The optimum temperature was lower than that for other chitinase reported from *A. fumigatus* YJ-407 (60°C), but it was higher than that of chitinase from *A. griseoaurantiacus* KX010988 (40°C) (Xia et al., 2001; Shehata et al., 2018). The thermostability of Δ30*Af*ChiJ was determined through preincubation for 60 or 90 min with different temperatures, from 25°C to 70°C, in the lack of substrate. The relative activity of Δ30*Af*ChiJ decreased slowly below 45°C, with a relative activity of 67% at 45°C for 60 min (48% at 45°C for 90 min). The activity of Δ30*Af*ChiJ dramatically decreased to about 24% at 50°C for 90 min, while more than 73% of the relative activity was maintained at 40°C for 60 min (Figure 3B). Therefore, Δ30*Af*ChiJ has higher activity and stability than most thermostability chitinases. So far, the thermostability of chitinases have had advantages for some utilizations where high temperature was required or could not be eliminated, for instance, the production of chitin oligomers and monomers could be used for aquaculture or material science. Therefore, Δ30*Af*ChiJ may demonstrate better utilization potential for such manufacture.

The optimal pH for Δ30*Af*ChiJ activity was shown in Figure 3C. The activity of Δ30*Af*ChiJ was maximal at pH 4, and it maintained high activity at pH 2.0 to 6.0, with relative activities of 36.42 and 36.24% at pH 2.0 and 6.0, respectively. There were have been a few reports on acid chitinases from *A. fumigatus*, and the majority of them exhibited optimal pH around 5.0. For instance, the optimum pH values of chitinase from *A. fumigatus* YJ-407, *A. fumigatus* CS-01, and *Acremonium sp.* YS2-2 were reported to be at 5.0, 5.0, and 6.0, respectively (Xia et al., 2001, 2009; Chung et al., 2019). Only the chitinase produced by *Streptomyces* and AMCase isolated from mouse stomach maintained the highest activity at pH 2.0 (Karthik et al., 2015; Du et al., 2021). The pH stability of Δ30*Af*ChiJ was shown in Figure 3D by pre-incubating substrate-free for 3 h at 30°C, using pH values from 1 to 11. The residual activity of Δ30*Af*ChiJ was the highest at pH 3.0 and was stabilization within the scope of pH 2–6 (relative activity >62%). It indicated that Δ30*Af*ChiJ is alkaline sensitive, while can be work well at acidic conditions. The majority of chitinases from marine fungus appeared medium high stability within the scope of pH 4–7 (Xia et al., 2009; Han et al., 2016; Wu Y et al., 2022). The Δ30*Af*ChiJ in this research was equivalent to the acidic chitinase from *A. fumigatus* CS-01, displaying stability at pH 2.0–6.0 for 3 h at 30°C, which was comparable to the chitinase from *Pyrus ussuriensis* (pH 2.5–6.0) (Xia et al., 2009; Han et al., 2016). On the other side, converting 20 g chitin to colloidal chitin requires approximately 10–12 L water to reach a neutral pH, while only 4–6 L is required to reach a pH of 4.0. Thus, it was saved half of the water consumption using an acidic chitinase of Δ30*Af*ChiJ. In summary, its high stability at low pH conditions and its lower water consumption requirements for processing indicate that Δ30*Af*ChiJ is a potential candidate for commercial and industrial applications (Ueda and Kurosawa, 2015).

### Effect of metal ions and chemical reagents on Δ30*Af*ChiJ activity

**FIGURE 4:**
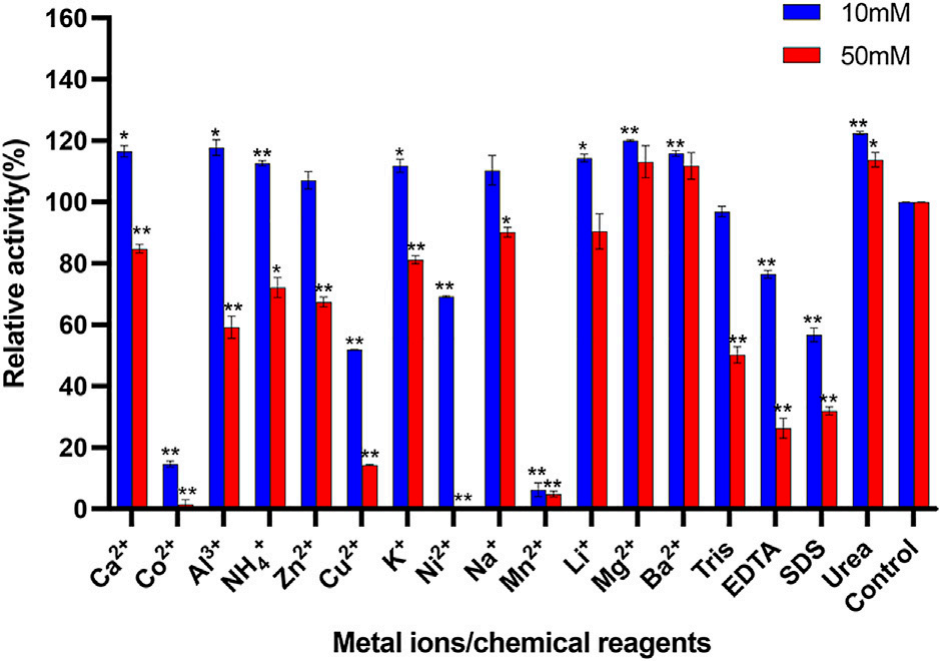
Effects of metal ions and chemical reagents on activity of Δ30AfChiJ. Untreated enzyme was used as the control, and the activity of this preparation defined as 100%. Experiments were carried out in triplicate, and standard errors are shown (mean ± SD). Asterisks indicate statistically significant differences from the control (Student’s t-test. *, *p* < 0.05; **, *p* < 0.01). Abbreviations: EDTA, ethylenediaminetetraacetic acid; SDS, sodium dodecyl sulfate.

The influences of metal ions and chemical reagents on the activity of purified Δ30*Af*ChiJ are shown in Figure 4. The Δ30*Af*ChiJ activity was inhibited within 10 mM Cu^2+^ or Ni^2+^, moreover, the activity dropped sharply within 50 mM concentration, even 50 mM Ni^2+^ completely no activity at all. It has been suggested that Cu^2+^ catalyzes the amino acids of cysteine to form intramolecular disulfide bonds or sulfenic-acid (Vieille and Zeikus, 2001) and to hinder chitinase activities from different microorganisms such as *Streptomyces sp.* and *Trichoderma harzianum* GIM 3.442 (Pradeep et al., 2014; Deng et al., 2019), this phenomenon may indicate the potential role of cysteines involved in the enzyme catalysis. At the same time, the enzymatic activity was mightily inhibited within both concentrations of Co^2+^, Mn^2+^, and SDS (relative activity <60%). Moreover, the activity of Δ30*Af*ChiJ was barely seen in the presence of Co^2+^ and Mn^2+^. This is because these heavy metal ions can deactivate proteins by disrupting the tertiary structure of enzymes (Liu et al., 2020). This is similar to the chitinase *MD*cht2 from *Musca domestica*, while enzymatic activities were thoroughly inhibited by Mn^2+^ and Fe^2+^ (Peipei et al., 2021). In this study, we also detected that the activity of Δ30*Af*ChiJ increased by adding Mg^2+^and Ba^2+^ (10 and 50 mM), which increased the enzymatic activity about 1.2-fold relative to control. Studies have found that metal ions serve as cofactors for enzyme activities, and after acting as ion or salt bridges between two adjacent amino acid residues, maintain rigid confirmation of enzymes (Kamran et al., 2015), this is close to ChiEn1 from *Coprinopsis cinerea*, where enzymatic activity is moderately increased by Mg^2+^and Ba^2+^ at 1 mM concentration (Niu et al., 2017). The effect of 10 and 50 mM urea on Δ30*Af*ChiJ is compatible with chitinase obtained from *Paenibacillus* UMBR 0002 (Liu et al., 2020). It is widely known that strong concentrations of urea denatures proteins, but we achieved the opposite result at 10 and 50 mM of urea. In addition, EDTA was a metal ion chelating agent, and the influence on enzyme activity demonstrated that Δ30*Af*ChiJ was greatly affected by the enzyme activity of metal ions. The effect of EDTA on Δ30*Af*ChiJ is similar to that on chitinase originated from Qinghai-Tibetan Plateau (Dai et al., 2021). Generally, these results provide a basis for which metals ions or chemical reagents to add for the commercial and industrial applications of Δ30*Af*ChiJ. The roles of the metal ions are related to substrate activation or electrostatic stabilization and played a vital role in biocatalysts by stabilizing the complexes of substrate-enzyme (Andreini et al., 2008).

### Effects of solvents on Δ30*Af*ChiJ activity

**FIGURE 5:**
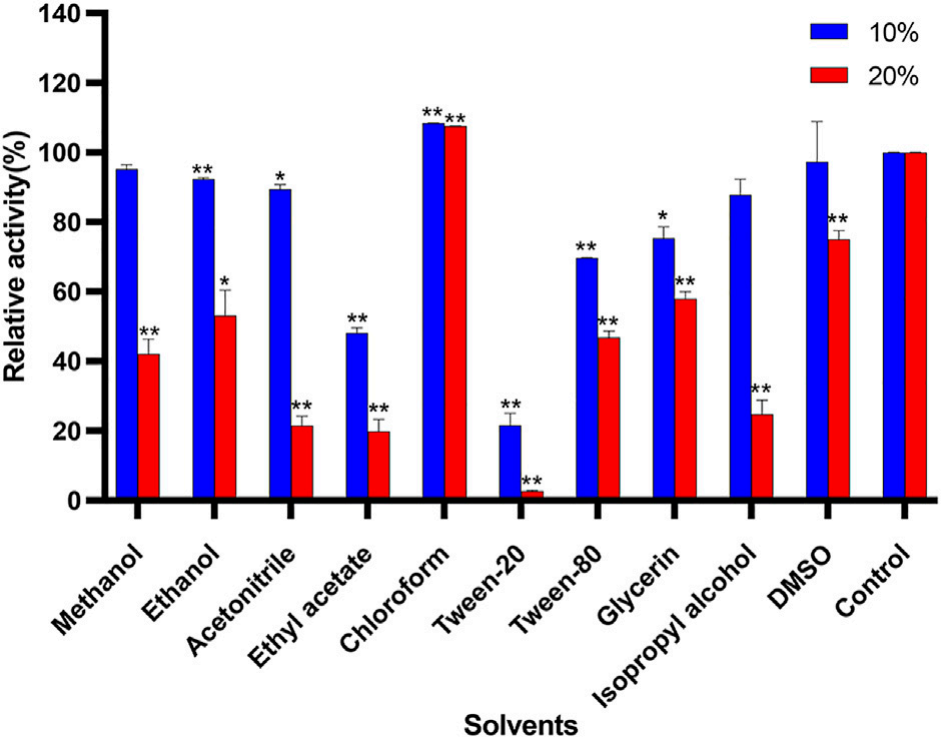
Effects of solvents on activity of Δ30*Af*ChiJ. Untreated enzyme was used as the control, and the activity of this preparation defined as 100%. Each data represents the mean of three independent experiments, standard errors are shown (mean ± SD). Asterisks indicate statistically significant difference, compared with the control (Student’s t-test. *, *p* < 0.05; **, *p* < 0.01). Abbreviation: DMSO, dimethylsulfoxide.

The effects of different organic compounds on the stability of Δ30*Af*ChiJ are displayed in Figure 5. In the presence of chloroform, there is a notedly increased (10 and 20%). The influence of chloroform on Δ30*Af*ChiJ is equivalent to the chitinase obtained from *Melghiribacillus* Nari2A (Mohamed et al., 2019). This indicates that the chitinase aqueous solution is mixed with chloroform, and the water molecules between the enzyme molecules are squeezed out by chloroform. Therefore, chloroform lowers the activation energy of the enzymatic reaction and promotes the progress of the enzyme reaction. On the other hand, for most organic solvents tested in his paper, including methanol, ethanol, acetonitrile, ethyl acetate, tween-20, tween-80, glycerin, and isopropyl alcohol, when using an ultimate concentration of 10%, the activity of Δ30*Af*ChiJ decreased, while the 20% organic solvents notedly inhibited the activity. Moreover, ethyl acetate and tween-20 strongly inhibited Δ30*Af*ChiJ activity (relative activity <50%) at both concentrations. In addition, Δ30*Af*ChiJ activity was not significantly affected by 10% DMSO, while relative activity moderately decreased (<70%) in the presence of 20% DMSO. In summary, the influences of different organic compounds in this paper on Δ30*Af*ChiJ activity approximated those of chitinases from *Aeromonas hydrophila* SBK1 (Halder et al., 2016). The property of Δ30*Af*ChiJ that was stable at an ultimate 10% concentration of most organic solvents suggested that the enzyme could be used in practical industrial applications (Mohamed et al., 2019). Thus, Δ30*Af*ChiJ might be a suitable candidate for commercial applications.

### Influence of NaCl concentration on Δ30*Af*ChiJ stability

**FIGURE 6:**
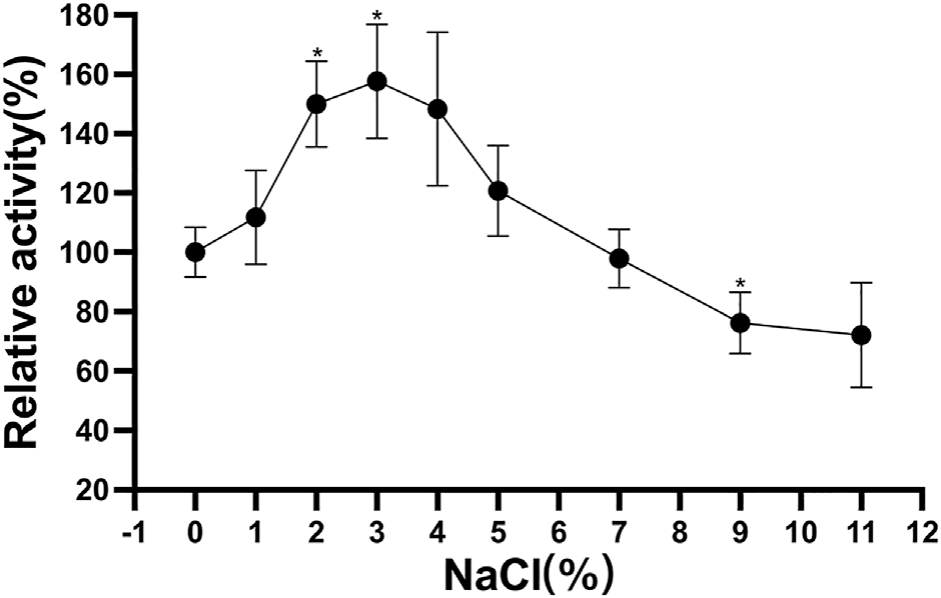
Effects of NaCl concentration on stability of Δ30*Af*ChiJ. Stability of Δ30*Af*ChiJ after incubation at various NaCl concentration at 30°C for 3 h. The residual activity of Δ30*Af*ChiJ was measured at 45°C and pH 4.0 with colloidal chitin as the substrate. The highest enzyme activity after preincubation at no NaCl concentration was defined as 100%. Each data represents the mean of three independent experiments, standard errors are shown (mean ± SD). Asterisks indicate statistically significant differences from the control (Student’s t-test. *, *p* < 0.05; **, *p* < 0.01).

The Δ30*Af*ChiJ showed highest relative activity of 157.71% at an optimal NaCl concentration of 3%. When it was incubated at 4°C after 3 h, the remained high activity (≥97.96%) was detected in the 1–7% concentration of NaCl. However, Δ30*Af*ChiJ activity decreased under the 11% NaCl, and its relative activity reached <72.19% (Figure 6). The concentration of NaCl in the tidal flat sediments of mangrove is approximately 3%; therefore, Δ30*Af*ChiJ could be used to hydrolyze chitin waste from oceanic and coastal environments (Liu et al., 2020).

### Substrate specificity of Δ30*Af*ChiJ

The specific activities of Δ30*Af*ChiJ (approximately 1 mg/ml) against different zymolyte are listed in Table 3. The specific activity to colloidal chitin was highest (110.47 mU/mL). The activity was 53.85 mU/mL when using β-chitin as a substrate. The relative activity was 48.74 ± 1.24% compared with colloidal chitin (100%). The activity against α-chitin was weak (3.82 mU/mL). No activity was detected toward cellulose microcrystalline, sodium carboxymethylcellulose (CMC-Na) and chitosan. Our results suggested that Δ30*Af*ChiJ preferred colloidal chitin to β-chitin and α-chitin. At the same time, colloidal chitin was the most suitable substrate for Δ30*Af*ChiJ. This suggested that the chitinase more easily accessed the treated colloidal chitin. For α-chitin, low Δ30*Af*ChiJ activity (3.8%) was detected, which likely entailed that α-chitin was too difficult to dissolve in the phosphate buffer. Chitinases belonging to GH family 18 possessed a substrate-assisted catalytic mechanism (van Aalten, 2021). The glycosidic bond protonated by the acid was a conservative glutamate, and the nucleophile is an oxygen of the N-acetyl group on the -1 sugar, producing an intermediate of oxazolinium ion. Due to this mechanism, a GlcNAc residue in the -1 subsite was obligatory for catalytic cleavage to appear (Hartl et al., 2012). This, it is reasonable to be unable to detect any activity for the chitosan, even if chitosan was dissolved in the optimum pH 4.0 of Δ30*Af*ChiJ. For cellulose microcrystalline and CMC-Na, the activity of Δ30*Af*ChiJ cannot be measured in our study, which was similar to chitinase from *T. harzianum* GIM 3.442 (Deng et al., 2019). Δ30*Af*ChiJ is a typical chitinase, and it can efficiently degrade colloidal chitin.

**TABLE 3:** Substrate specificity of Δ30*Af*ChiJ.

Substrate	Specific activity^a^(mU/ml)	Relative activity^a^ ^,^ ^b^(100%)
Colloidal chitin	110.47 ± 0.07	100 ± 0.07
β-Chitin	53.85 ± 1.37	48.74 ± 1.24
α-Chitin	3.82 ± 2.91	3.45 ± 2.64
Cellulose Microcrystalline	0	0
CMC-Na	0	0
Chitosan	0	0

a Experiments were conducted three times and standard errors are reported.

b Relative enzyme activity calculation using colloidal chitin as the reference value (100%). Abbreviations: CMC-Na, carboxymethyl cellulose sodium salt.

### TLC and HPLC analysis of the hydrolysis product

**FIGURE 7:**
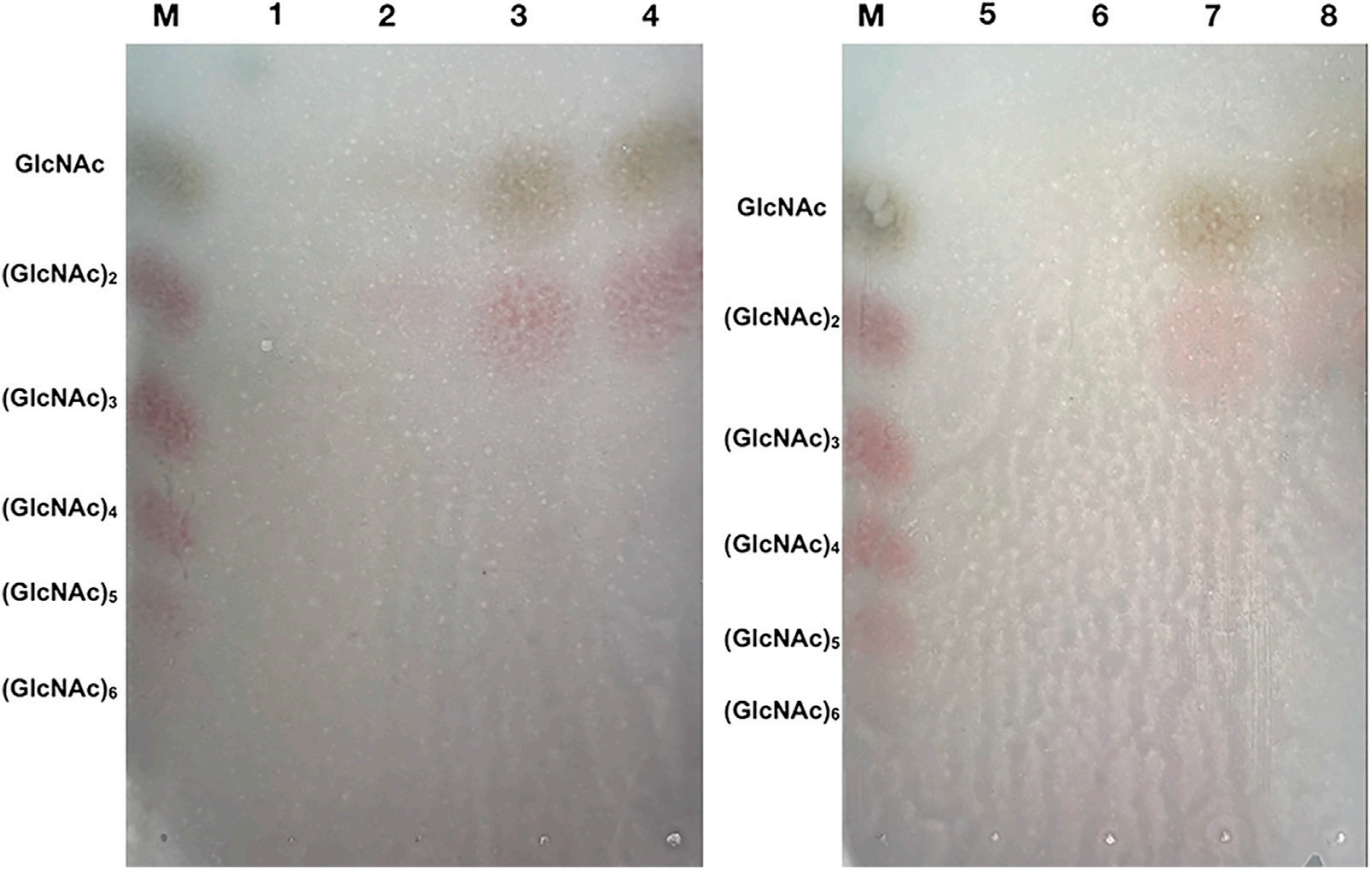
TLC analysis of the degradation products of Δ30*Af*ChiJ. M, Standards of chitin oligosaccharides with degrees of polymerization between 1 and 6; 1 and 5, The degradation products of inactivation Δ30*Af*ChiJ was used to be control; 2–4, The degradation products of Δ30*Af*ChiJ with colloidal chitin for 2, 4, and 8 h; 6–8, The degradation products of Δ30*Af*ChiJ with colloidal chitin for 1, 3, and 6 h. Developing solvent: n-butanol, acetic acid and aqueous (2:1:1). Color reagents: 1 g diphenylamine, 1 ml aniline, 5 ml 85% phosphoric acid, and 50 ml acetone.

**FIGURE 8:**
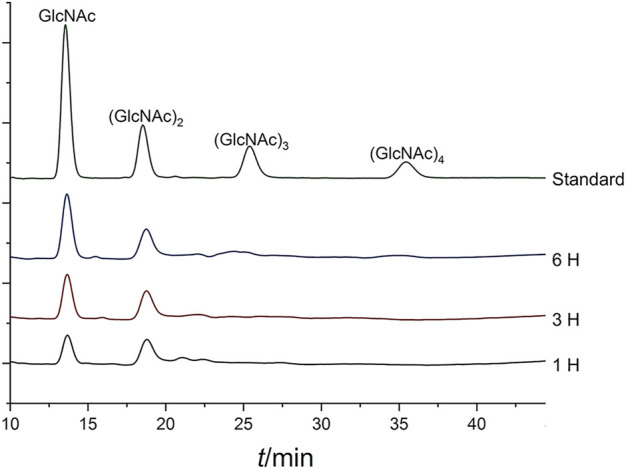
HPLC analysis of the degradation products of Δ30*Af*ChiJ. Colloidal chitin was degraded by Δ30*Af*ChiJ at 45°C and pH 4.0 for different lengths of time (1, 3, and 6 h). A refractive index detector and a Phenomenex Luna NH_2_ column (250 mm × 4.6 mm) were used to analyze 10 microliters supernatant of the reaction mixtures. The mobile phase was acetonitrile: water (75:25, v/v) with a flow rate of 0.6 ml/min, the column temperature was 45°C.

In this study, we determined a mixed product including (GlcNAc)_2_ and GlcNAc during the degradation of colloidal chitin. As shown in Figure 7, (GlcNAc)_2_ and GlcNAc can be observed at 2 h by TLC assay, and the amount of product was gradually accumulated with the increase of reaction time. However, we could not detect any products at 1 h, and we presumed that the amount of reaction products at 1 h was below the minimum detection limit of the TLC method. Therefore, the colloidal chitin hydrolysate by Δ30*Af*ChiJ at 1, 3, and 6 h was detected using HPLC. The results appeared that the products of (GlcNAc)_2_ and GlcNAc in the reaction of Δ30*Af*ChiJ and colloidal chitin for 1 h were low. At the same time, the peak heights of (GlcNAc)_2_ and GlcNAc were nearly the same (Figure 8). This shows that the yields of (GlcNAc)_2_ and GlcNAc were basically comparable in the early stage of the enzyme-substrate reaction. The amount of GlcNAc increased obviously after 3 h, while the amount of (GlcNAc)_2_ increased slightly, which suggested that Δ30*Af*ChiJ could hydrolyze colloidal chitin to obtain GlcNAc as the major product and (GlcNAc)_2_ as a minor product. Meanwhile, the Δ30*Af*ChiJ retains stability and activity for 6 h, which could be an appropriate reaction time to obtain GlcNAc.

Chitinases can be divided into two major groups: endochitinase and exochitinase. Endochitinase hydrolyzes chitin randomly at internal sites, generating low molecular mass oligosaccharide, such as the forms (GlcNAc)_2_, (GlcNAc)_3_, and (GlcNAc)_4_ forms. Exochitinase can be classified into two subcategories: chitobiosidases progressively liberate (GlcNAc)_2_ beginning at the chain end of chitin, and β-N-acetylglucosaminidase cleave the (GlcNAc)_2_ to generate monomers of GlcNAc (Sahai and Manocha, 1993; Dahiya et al., 2006). The chitinase Δ30*Af*ChiJ from *A. fumigatus* df673 may be an exochitinases, which represents both chitobiosidases and β-N-acetylglucosaminidase activity. In our study, GlcNAc and (GlcNAc)_2_ were detected to be hydrolysis products from colloidal chitin in the whole phase, and (GlcNAc)_2_ was detected, suggesting that the chitinase could cleave preferentially terminal glycosidic bonds, thereby demonstrating a chitobiosidases activity. Interestingly, (GlcNAc)_2_ was further hydrolyzed to GlcNAc, it was suggested that Δ30*Af*ChiJ had β-N-acetylglucosaminidase activity. The hydrolysis mechanism and processing of Δ30*Af*ChiJ is close to the chitinase of the Baozhu pear from *Pyrus ussuriensis Maxim* and PbChi74 from *Paenibacillus barengoltzii* (Fu et al., 2014; Han et al., 2016). These cleave colloidal chitin to obtain GlcNAc as the major product and possess two chitinase activities (exochitinase and β-N-acetylglucosaminidase activities). The majority of the 18 other GH family exochitinases exhibit a proceeding mode of action to produce (GlcNAc)_2_ as the main end product, while they cannot further degrade (GlcNAc)_2_ to GlcNAc (Horn et al., 2006). Meanwhile, the chitinase *Cm*Chi1 from *meiyuanensis* SYBC-H1 has shown the ability of endochitinase and chitobiosidase with β-N-acetylglucosaminidase activities (Zhang et al., 2018). In general, the biofacturing of GlcNAc is a valuable “green” method, and the special properties of Δ30*Af*ChiJ may make chitinase a suitable candidate for the converting chitin into GlcNAc. Therefore, the hydrolysis activity of Δ30*Af*ChiJ facilitates the commercial production of GlcNAc from chitin. Nevertheless, the catalytic mechanism of Δ30*Af*ChiJ hydrolyzed chitin requires further research.

### The β-N-acetylglucosaminidases activity assay

The yield of GlcNAc increased obviously with colloidal chitin hydrolysis by Δ30*Af*ChiJ for 6 h (Figure 8), so we deduced Δ30*Af*ChiJ may have β-N-acetylglucosaminidases activity. 4MU-GlcNAc was chosen to mimic (GlcNAc)_2_. The β-N-acetylglucosaminidase activity of Δ30*Af*ChiJ was measured by cleavage of 4MU-GlcNAc substrates and released 4MU fluorescent monomers. The fluorescence value was 264.5, obtained by a Fluorescence Spectrophotometer monitored (excitation 360 nm, emission 460 nm). The β-N-acetylglucosaminidase activity of Δ30*Af*ChiJ was 4.46 mU/mL with the standard curve calculation, and the specific activity of Δ30*Af*ChiJ was 5.57 mU/mg, all monitored under the specified assay conditions. This result suggests that Δ30*Af*ChiJ has β-N-acetylglucosaminidase activity; it is first demonstrated that β-N-acetylglucosaminidase activity existed in the *Aspergillus* family chitinases. There are many *Aspergilli* family chitinases with exochitinase activity, but little β-N-acetylglucosaminidase activity has been verified (Kuranda and Robbins, 1987; Rush et al., 2010).

### Alignment and molecular docking simulation

**FIGURE 9:**
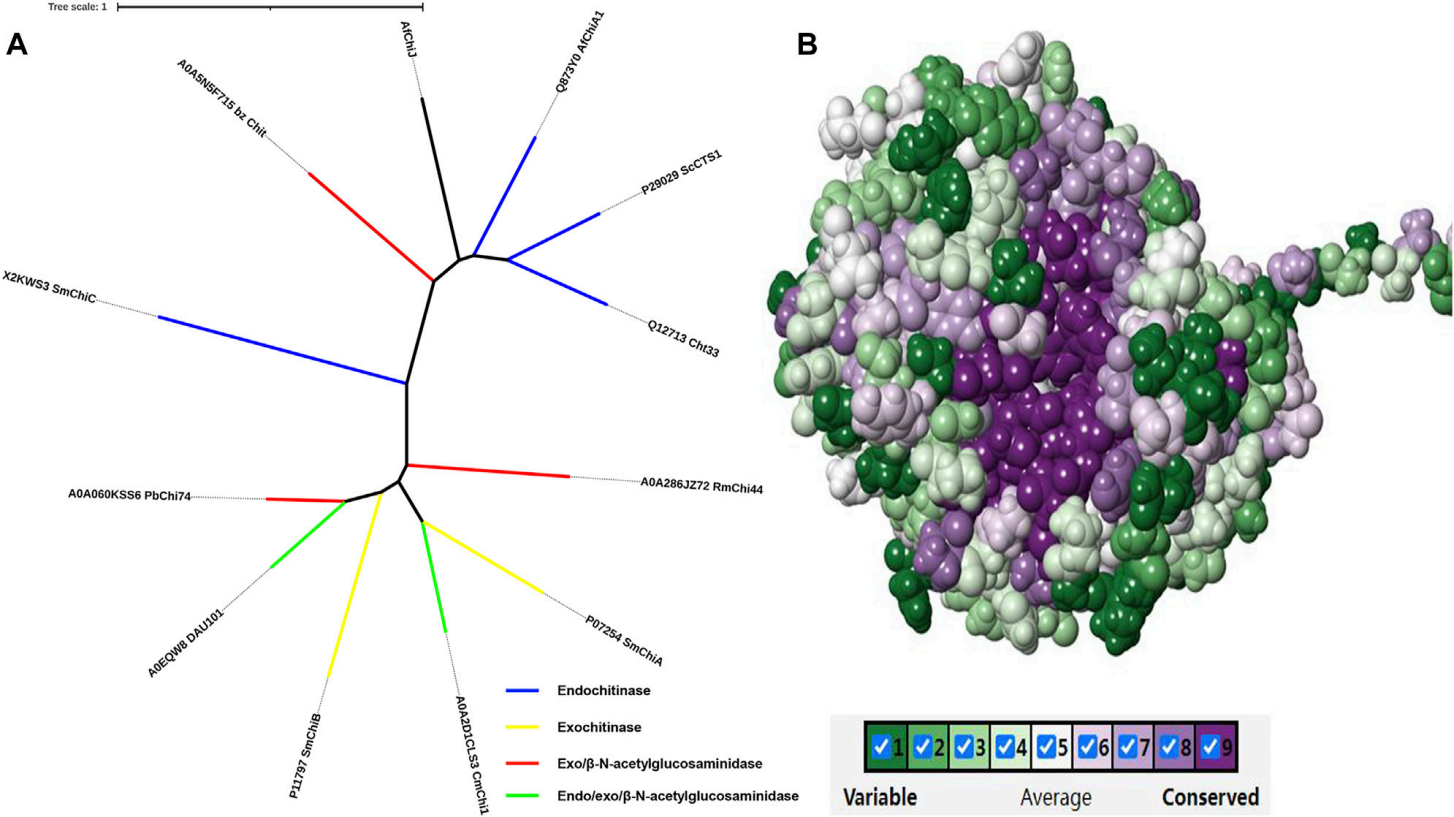
Phylogenetic tree analysis and evolutionary analysis of *Af*ChiJ. **(A)** The phylogenetic tree analysis of *Af*ChiJ. The endochitinases are shown in blue, exochitinases are shown in yellow, exo and β-N-acetylglucosaminidases are shown in red, endo/exo and β-N-acetylglucosaminidases are shown in green. Tree scale: 1 substitution per nucleotide position. **(B)** The evolutionary conservation is determined based on the structure and multiple sequence alignment. The amino acid residues are colored ingredient representing the conservation grades from the most variable (turquoise) to the most conserved (maroon) obtained with ConSurf program.

Following the phylogenetic tree analysis of *Af*ChiJ (Figure 9A), *Af*ChiJ was compared to 11 sequences with different properties which were characterized in Uniport. Among of these, an endochitinase from *A. fumigatus* ChiA1 (Uniport id Q873Y0) has a homology (36.9%) with *Af*ChiJ, another chitinase with exochitinase and β-N-acetylglucosaminidase properties from the Baozhu pear (Uniport id A0A5N5F715) has a homology (29.24%) with respect to *Af*ChiJ. Due to the lack of sufficiently characterized chitinases from filamentous fungi, it is difficult to determine the endo, exo, or β-N-acetylglucosaminidase properties of *Af*ChiJ based the homologous sequence alignment. Thus, we infer that it is a GH18 chitinase that belongs to the fungal/plant class chitinase (class III) family. Comparing to available structures of GH18 family member enzymes indicates that *Af*ChiJ is most similar to *A. fumigatus* ChiA1 (PDB id 2XTK) and *S. cerevisiae* CTS1 (PDB id 2UY4), with an RMSD (root mean square derivation) of 1.6 Å (286 C*α* atoms) and 1.8 Å (278 C*α* atoms), respectively. In the meantime, some different conformations occur in the connecting loops, most of which harbor insertions/deletions. Herein, we use the ConSurf server to identify the functional regions of *Af*ChiJ (Glaser et al., 2003). It is also demonstrated that the flexible region of the *Af*ChiJ (colored turquoise) exist in the connecting loops (Figure 9B). However, these flexible regions endow *Af*ChiJ with substrate specificity and catalytic properties.

**FIGURE 10:**
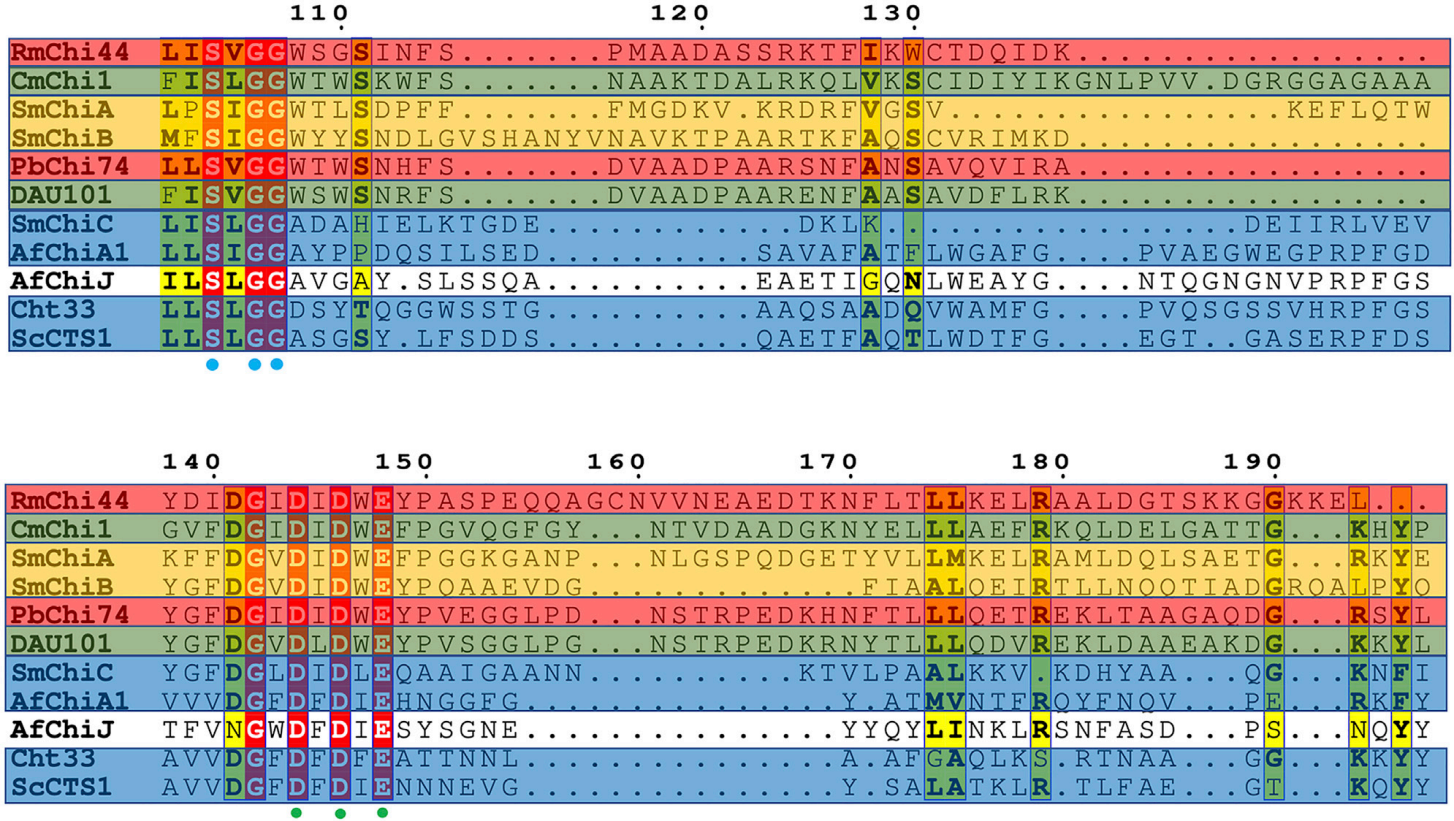
Alignment of amine acid sequence homology. Structure-based sequence alignment of 11 homology proteins. Residue numbers are given shaded by sequence similarity between the 11 enzymes shown (red = identical, yellow = chemically similar residues). Residues DXDXE are highlighted by green filled circles. Residues SXGG are highlighted by blue filled circles. Endochitinases are shown in blue background, exochitinases are shown against a yellow background, exo and β-N-acetylglucosaminidases are shown against a red background, endo/exo and β-N-acetylglucosaminidases are shown against a green background.

**FIGURE 11:**
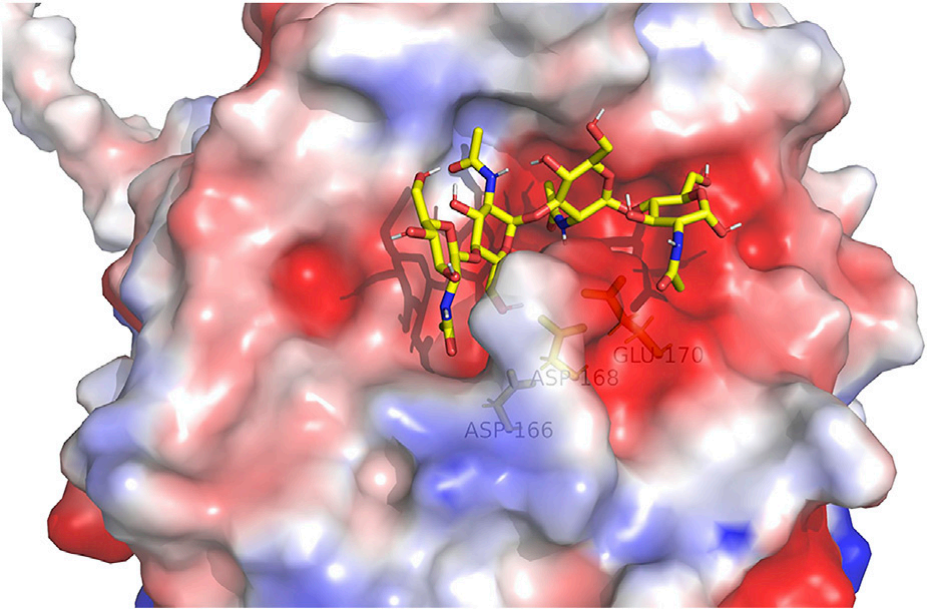
(GlcNAc)_4_ by molecularly docking with the *Af*ChiJ. Substrates with the active sites channel were simulated by docking (GlcNAc)_4_ with the AlphaFold2 model of *Af*ChiJ. Molecular docking revealed that the (GlcNAc)_4_ binding cavity of *Af*ChiJ contains four glycosyl binding sites, and that the predicted catalytic residue of *Af*ChiJ, Glu 170, is spatially close to the glycosidic bond between −1 and +1.

For the alignment of the amino acid sequence of the 11 homology proteins (Figure 10), analysis revealed that *Af*ChiJ contain the catalytic signature motif of GH 18, DxDxE (residues 144–148), and a chitin-binding motif, SxGG (residues 104–107). The glutamate 144 residue is considered a proton donor, and the aspartate 148 residue play a prime role in supporting the acetamido group in it nucleophilic attack and supporting the forming a positive oxazolinium ion charge (Bokma et al., 2022). Substrates with the active sites groove were mimicked by docking (GlcNAc)_4_, using the model of *Af*ChiJ simulated by AlphaFold2 (Figure 11). It was found that the active pocket formed a shallow and open groove. The catalytic motif DxDxE (residues 166–170) is located between β4-α4. It was found that the (GlcNAc)_4_ binding pocket of *Af*ChiJ contained four glycosyl binding sites using molecular docking, and that the predicted catalytic residue of *Af*ChiJ, Glu 170 is space-based near to the glycosidic bond among subsite −1 and subsite +1.

## Conclusion

In summary, we successfully cloned and expressed chitinases *Af*ChiJ, Δ19*Af*ChiJ, and Δ30*Af*ChiJ using *E. coli* BL21 (DE3). It is worth mentioning that Δ30*Af*ChiJ was successfully purified. The molecular weight of enzyme Δ30*Af*ChiJ was about 35 kDa and the hydrolytic activity of colloidal chitin was best at pH 4 and 45°C. TLC and HPLC analysis manifested that GlcNAc was the main hydrolysates of colloidal chitin. Furthermore, β-N-acetylglucosaminidase activity of Δ30*Af*ChiJ was measured by the cleavage of 4MU-GlcNAc substrates, releasing 4MU fluorescent monomers. Finally, it was verified that Δ30*Af*ChiJ represents both chitobiosidases and β-N-acetylglucosaminidase activities. A chitobiosidases showing β-N-acetylglucosaminidase activities from *Aspergillus* species was firstly reported. In addition, better enzymatic activity and stability were inspected over a wide scope of pH and temperature. The existence of Ca^2+^, Mg^2+^, and chloroform notably increased the activity of chitinase. The hydrolytic properties and great environmental adaptions made the Δ30*Af*ChiJ a potential candidate for green industrial transformation chitin debris into monomer of chitin. Generally, Δ30*Af*ChiJ might be applied for chitinous biomass high-value added utilization in the future.

## Data Availability

The datasets presented in this study can be found in online repositories. The names of the repository/repositories and accession number(s) can be found in the article/Supplementary Material.
